# Supplementary material for: Generation of High-Yield, Functional Oligodendrocytes from a c-myc Immortalized Neural Cell Line, Endowed with Staminal Properties
Source: Int J Mol Sci. 2021 Jan 23;22(3):1124. doi: 10.3390/ijms22031124 (PMC7865411; doi:10.3390/ijms22031124)
Supplement: Supplementary file 1 [file ijms-22-01124-s001.pdf]

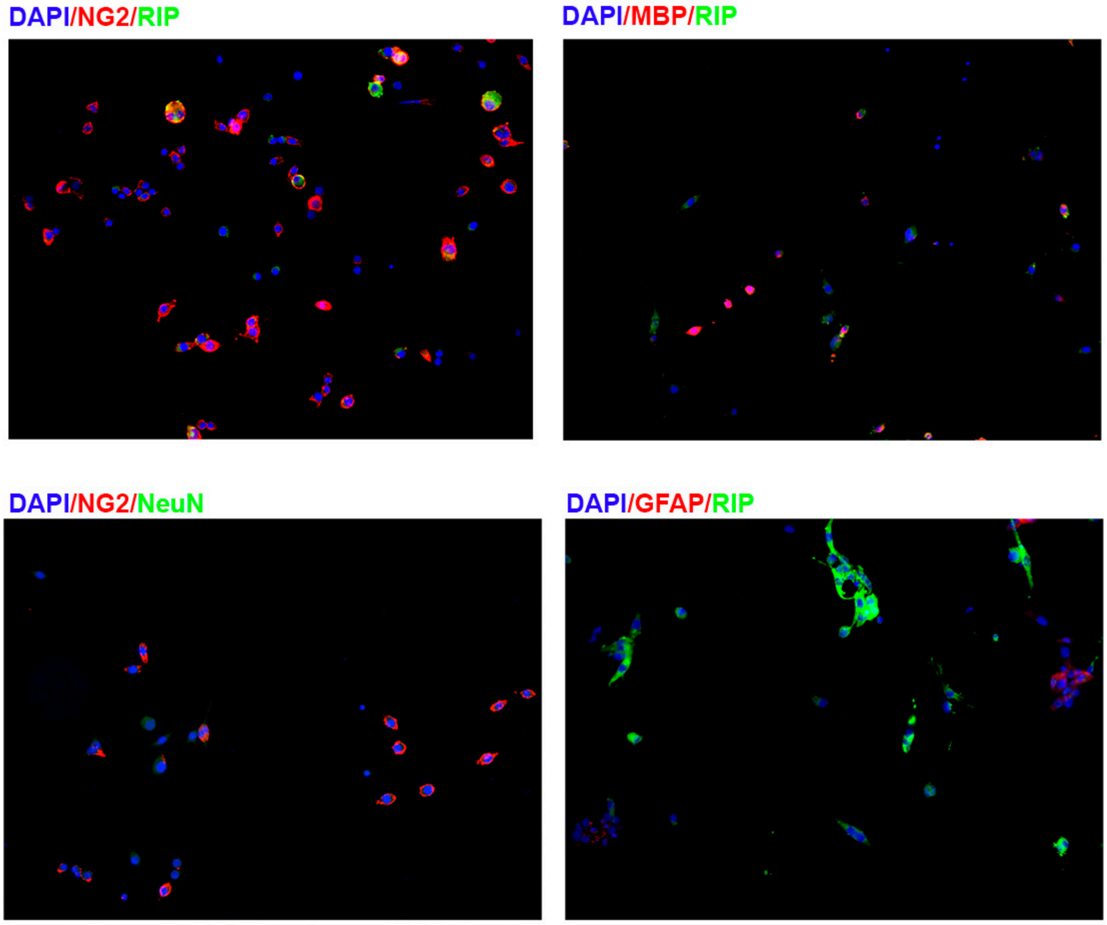

Supplementary Figure S1. Immunofluorescence analysis of the NG2 (red), RIP (green), MBP (red), NeuN (green), GFAP (red) and DAPI nuclei (blue) in diff-Ns at day 3. Magnification 20x.

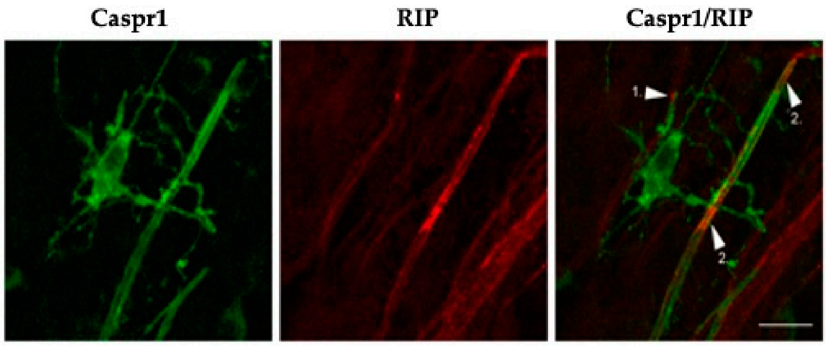

Supplementary Figure S2. A Rip positive oligodendrocyte (green) myelinating an axon (red) in a 4 weeks old mixed cerebellar culture, where all cell types of the rat cerebellum are present. At the point of interaction between the axon and the oligodendrocyte, Caspr 1 (in red) accumulates (arrows). The Caspr1 is later pressed in the front of the growing myelin sheath and when myelination is finalized, Caspr 1 is accumulated in the Node of Ranvier.
